# Intact painful sensation but enhanced non-painful sensation in individuals with autistic traits

**DOI:** 10.3389/fpsyt.2024.1432149

**Published:** 2024-07-09

**Authors:** Huiling Qian, Min Shao, Zilong Wei, Yudie Zhang, Shuqin Liu, Lu Chen, Jing Meng

**Affiliations:** ^1^ Research Center for Brain and Cognitive Science, Chongqing Normal University, Chongqing, China; ^2^ Key Laboratory of Applied Psychology, Chongqing Normal University, Chongqing, China

**Keywords:** pain, non-pain, autism, autistic traits, ERP

## Abstract

Somatosensory abnormalities are commonly recognized as diagnostic criteria in autism spectrum disorder (ASD), and may also exist in individuals with autistic traits. The present research included two studies to explore the painful and non-painful sensation and their cognitive-neurological mechanisms of individuals with autistic traits. Study 1 included 358 participants to assess the relationship between autistic traits and pain/non-pain sensitivities using questionnaires: the Autism Spectrum Quotient (AQ), the Pain Sensitivity Questionnaire, and the Highly Sensitive Person Scale, respectively. Study 1 found that autistic traits were positively correlated with non-pain sensitivity, but not associated with pain sensitivity. Study 2 recruited 1,167 participants whose autistic traits were assessed using the AQ. Subsequently, thirty-three participants who scored within the top 10% and bottom 10% on the AQ were selected into High-AQ and Low-AQ groups, respectively, to explore the cognitive-neural responses of individuals with autistic traits to both painful and non-painful stimuli with event-related potential (ERP) technology. Results of Study 2 showed that the High-AQ group showed higher intensity ratings, more negative emotional reactions, and larger N1 amplitudes than the Low-AQ group to the non-painful stimuli, but no difference of response to the painful stimuli was found between High-AQ and Low-AQ groups. These findings suggest that individuals with autistic traits may experience enhanced non-painful sensation but intact painful sensation.

## Introduction

Autism spectrum disorder (ASD) is a complex neurodevelopmental disorder characterized by challenges in social interaction and communication skills, as well as restricted interests and repetitive behaviors (1). In addition, somatosensory abnormalities have been included as a diagnostic criterion of ASD within the Diagnostic and Statistical Manual of Mental Disorders, 5th Edition (*DSM-5;* American Psychiatric Association, 2013).

Previous studies have indicated that the quantifiable autistic traits included in the ASD core deficits are continuously distributed in typically developing individuals (2). The Autism Spectrum Quotient (AQ) (3) has been used to estimate autistic traits in both ASD individuals and typically developing individuals. Individuals with ASD were usually identified by extremely high AQ scores compared with the general population (4). With this questionnaire, typically developing people with high AQ scores (i.e., those exhibiting the top 10% of AQ scores) (5, 6), who do not fully match the ASD clinical diagnostic criteria, can be identified as individuals with autistic traits (7, 8). Individuals with autistic traits often manifest deficits in social interactions in visual (5, 9–11) and auditory (6, 12, 13) modalities, and may also present alterations in sensory processing in other modalities.

Although anomalous somatosensory experiences of the skin (including painful and non-painful sensation) in individuals with ASD are recognized as a core phenotypic hallmark of autism (14), many previous studies did not find a difference between individuals with ASD and typically developing individuals in their responses to painful stimuli (15–17). In addition, the non-painful sensation in individuals with ASD are heterogeneous: some studies have reported their hyperresponsiveness to non-painful stimuli (18, 19), while others have not (20). Furthermore, the somatosensory experiences of individuals with autistic traits are still in the initial stages of exploration.

In order to precisely investigate the painful and non-painful sensation of individuals with autistic traits, this study employs event-related potential (ERP) technology. ERP components, specifically the N1 and P2, are crucial for understanding these sensory experiences. The N1 component, typically associated with the initial sensory processing of stimuli, reflects the primary cortical response to both painful and non-painful sensory inputs (21, 22). The later P2 component is indicative of higher-level processing, involving affective-motivational aspects of sensory experience (23, 24), which can provide deeper insights into how individuals with ASD process emotional aspects of these stimuli.

Some studies have explored painful sensation in individuals with autistic traits. For instance, Zhang et al. (25) employed the AQ questionnaire to quantify autistic traits and randomly selected two subsets of groups (High-AQ and Low-AQ groups) from the participants with the highest and lowest 10% AQ scores, respectively. This study investigated the relationship between autistic traits and the painful sensation through ERP technology, but did not find a significant difference in the response to painful stimuli between the High-AQ and Low-AQ groups. Nevertheless, previous study did not examine the non-painful sensation in individuals with autistic traits. Therefore, this research aims to use both painful and non-painful stimuli to further investigate the cognitive and neural mechanisms of sensory experiences of the skin in individuals with autistic traits.

Our paper includes two studies to explore the painful and non-painful sensation in individuals with autistic traits. Study 1 aims to explore the correlation between autistic traits and pain/non-pain sensory sensitivity through a large-scale questionnaire survey. Study 2 uses the AQ questionnaire to categorize participants into individuals with autistic traits (High-AQ group) and control group (Low-AQ group), aiming to explore the painful and non-painful sensation, as well as their cognitive-neurological mechanisms in individuals with autistic traits using ERP technology. Based on the Enhanced Perceptual Functioning model (26), which suggests that individuals with ASD would experience elevated perceptual sensitivity because of enhanced perceptual discrimination and increased attention to sensory details, we hypothesized that individuals with autistic traits might experience increased non-painful sensation. However, considering the critical evolutionary role and the prominence of pain, we hypothesized that individuals with autistic traits might maintain intact painful sensation.

## Methods

### Study 1: the relationship between autistic traits and pain/non-pain sensitivity

#### Participants

An a priori power analysis using G*Power 3 (27) revealed that 112 participants were required to reach a good statistical power of 0.9 to detect an absolute correlation coefficient of 0.3 with an alpha value of 0.05. Following this, a total of 358 adults (221 females) aged 18 years − 25 years, with means (*M*) = 19.39 years, standard deviations (*SD*) = 1.68 years, from the Chongqing Normal University, China, were recruited in this study. All participants were right-handed and had normal or corrected-to-normal vision. No participant reported any medical condition associated with acute or chronic pain, neurological diseases, psychiatric disorders, or current use of any medication. All participants gave their written informed consent before the experiment according to the Declaration of Helsinki. All experimental procedures were approved by the local research ethics committee.

#### Materials and measures

##### Autistic traits

The Mandarin version (28) of the AQ questionnaire (3), which is considered a reliable instrument for measuring autistic traits in both clinical and non-clinical populations was used in this study. The AQ questionnaire comprises 50 self-report items and measures autistic traits across five subscales: imagination, social skill, communication, attention switching, and attention to detail. Participants were asked to indicate their level of agreement with each item (e.g., “I prefer to do things with others rather than on my own”) using a 4-point scale (1 = *definitely disagree*, 2 = *slightly disagree*, 3 = *slightly agree*, 4 = *definitely agree*). Total AQ scores range from 50 to 200 (28), with higher scores indicating higher levels of autistic traits. In this study, Cronbach’s α of the AQ was 0.662.

##### Pain sensitivity

The Chinese version of the Pain Sensitivity Questionnaire (29), which has proven to be a valid instrument to evaluate pain sensitivity among healthy adults as well as patients with chronic pain (30). The Pain Sensitivity Questionnaire comprises 17 self-report items (e.g., “Imagine you trap your finger in a drawer.”) and measures pain sensitivity in two subscales: moderate pain and minor pain. Participants were asked to rate how painful this situation would be for them on a 11-point Likert scale (0 = *not painful at all*, 10 = *worst pain imaginable*). Three items (corresponding to items 5, 9, 13 of the Pain Sensitivity Questionnaire) described normally non-painful situations (e.g., “Imagine you take a shower with lukewarm water.”). Thus, the Pain Sensitivity Questionnaire score reflects an average of 14 items, with higher scores indicating higher pain sensitivity. In this study, Cronbach’s α of the Pain Sensitivity Questionnaire was 0.903.

##### Non-pain sensitivity

The Mandarin version (31) of the Highly Sensitive Person Scale (32), which is considered a reliable instrument for measuring individual differences mainly in non-painful sensory sensitivity was used in this study. The Highly Sensitive Person Scale, consisting of 27 items which represents physiological reactivity to stimuli in the environment as well as more subtle reactivity (e.g., “Are you easily overwhelmed by strong sensory input? “), with higher scores indicating higher sensory sensitivity. The questionnaire has seven self-report items answered using a 7-point Likert scale (1 = *not at all*, 7 = *extremely*). In this study, Cronbach’s α of the Highly Sensitive Person Scale was 0.915.

#### Data analysis

Data analyses were performed using MATLAB R2016a (MathWorks, Natick, MA, USA), including descriptive statistics, internal reliability (α) estimates, and correlation analyses. Pearson product-moment correlation analysis was used to examine the relationship among Autistic Traits, Pain Sensitivity, and Non-pain Sensitivity. Tests of normality revealed that the study variables showed no significant deviation from normality (e.g., Skewness < |3.0| and Kurtosis < |10.0|) (33, 34).

To examine the variation in scores on each questionnaire in individuals with autistic traits, two subsets of 35 participants, those scoring within the top 10% and bottom 10% on the AQ (9, 13) from the total of 358 adults were randomly selected and divided into High-AQ (*n* = 35, 17 females, age: 19.66 years ± 1.03 years) and Low-AQ (*n* = 35, 16 females, age: 19.77 years ± 1.40 years) groups. Then, an independent samples *t*-test was conducted to compare differences on each questionnaire between the two groups.

### Study 2: cognitive-neural responses to painful and non-painful stimuli in individuals with autistic traits

#### Participants

According to previous studies of autistic traits (7, 12, 35, 36), a total of 1167 university students at the Chongqing Normal University, China, aged 18 years − 26 years (*M* = 21.73 years, *SD* = 1.36 years) were recruited to complete the Mandarin version (28) of the AQ questionnaire (3), which was used to measure their autistic traits.

An a priori power analysis using G*Power 3 (27) indicated that a sample size of 27 per group was needed to attain a statistical power of 0.95 for detecting median-sized effects (f = 0.25) with an alpha level of 0.05 in a 2 × 2 within-between repeated measures analysis of variance (ANOVA). Following this, thirty-three participants (17 females) were randomly selected from the 10% of students with the highest AQ scores and were identified as the High-AQ group. A further thirty-three participants (16 females) were randomly selected from the 10% of students with the lowest AQ scores, and were identified as the Low-AQ group (12, 36). Ages and AQ scores of each group are summarized in **Table 1**. Criteria for inclusion were: Normal or corrected-to-normal vision, no medical condition associated with acute or chronic pain, neurological diseases, psychiatric disorders, or current use of any medication. All participants provided written informed consent before participating in the experiment. The study was approved by the Chongqing Normal University Research Ethics Committee, and all procedures were performed under ethical guidelines and regulations.

**Table 1:** Demographic characteristics of the High-AQ and Low-AQ groups.

Group	Age (years)			AQ scores		
	*M* ± *SD*	*t*	*p*	*M* ± *SD*	*t*	*p*
High-AQ	19.88 ± 1.08	-0.09	0.926	30.64 ± 1.75	39.09	< 0.001
Low-AQ	19.91 ± 1.51			13.06 ± 1.90		

AQ, Autism Spectrum Quotient. Statistical results were obtained using independent sample t-tests between the High-AQ and Low-AQ groups.

#### Stimuli

Stimuli were delivered through electrodes placed on the dorsum of the left hand using a constant current stimulator (SXC-4A, Sanxia Technique Inc., China). Based on previous studies (37–41), the present study set the calibrated electrical current for stimuli at 500 μA (non-painful stimuli) and 1200 μA (painful stimuli) for female participants, and 1000 μA (non-painful stimuli) and 2755 μA (painful stimuli) for male participants.

#### Procedure

**Figure 1:**
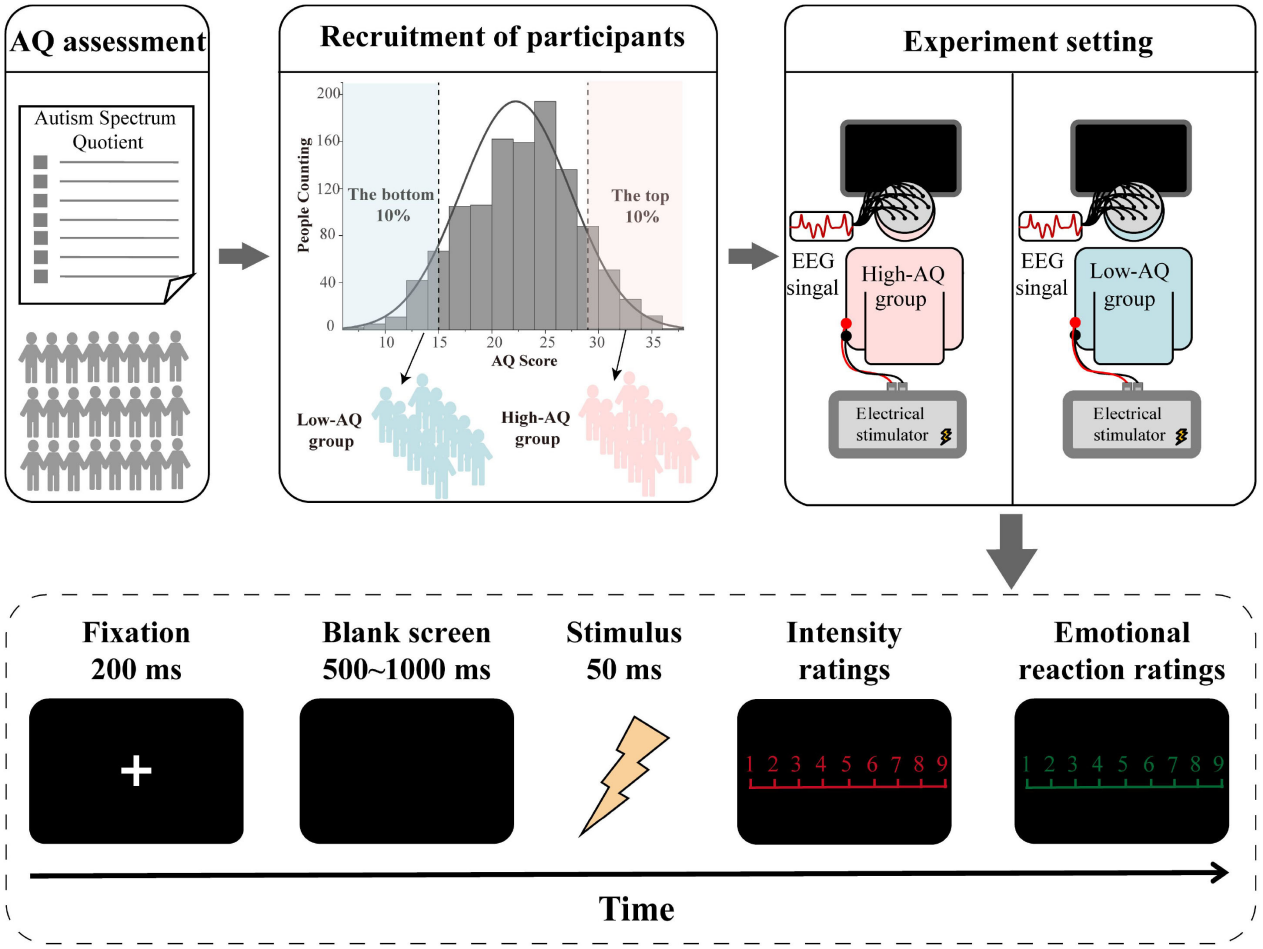
Flowchart describing the experimental design.

The participants were seated in a quiet room with an ambient temperature of about 22°C to complete the experiment while electroencephalography (EEG) recordings were documented. For this experiment, the stimuli were presented in random order. Stimuli presentation was controlled using the E-Prime (3.0) program. At the start of each trial of the experiment, a 200 ms fixation cross was presented on a black screen, followed by a stimulus after 500 ms − 1,000 ms. The stimulus lasted for 50 ms, and participants were instructed to respond as accurately and quickly as possible by pressing a key (either “1” or “2”) to indicate whether the stimulus was painful or non-painful. Key-pressing was counterbalanced across participants to control for potential order effects. The intensity ratings (1 = *no sensation*, 4 = *pain threshold*, 9 = *unbearable pain*) of the stimulus and the subjective emotional reaction ratings (1 = *very happy*, 9 = *very unhappy*) to the stimulus were then rated using 9-point scales. The inter-trial interval was 4,000 ms − 6,000 ms. The study comprised 40 trials, including 20 painful and 20 non-painful stimuli. The experimental procedure is illustrated in **Figure 1**.

#### EEG recording and data analyses

The EEG data were recorded from 64 scalp sites using tin electrodes mounted on an actiCHamp system (Brain Vision LLC, Morrisville, NC, US). The electrode on the frontal mastoid was used as a recording reference, and the one on the medial frontal aspect was used as a ground electrode. All electrode impedances remained below 5 kΩ.

EEG data were pre-processed and analyzed via MATLAB R2016a and the EEGLAB toolbox (42). Continuous EEG signals were band-passed, filtered (0.1 Hz − 40 Hz), and segmented using a 1,200 ms time window. ERPs at each electrode were re-referenced to the algebraically computed average of the left and right mastoids before further analysis. Time windows of 200 ms before and 1,000 ms after the onset of stimuli were extracted from the continuous EEG. EEG epochs were baseline-corrected by a 200 ms time interval before stimuli onset. Electro-oculographic artifacts were corrected with an independent component analysis algorithm (43).

After confirming scalp topographies in both the single participant and group-level ERP waveforms, as well as on the basis of previous studies (41, 44–47), the dominant ERP components were identified, including N1 and P2. These components have been strongly associated with the processing of painful and non-painful stimuli (48–53). Amplitudes of N1 were measured at the right-central electrodes (Fz, F2, F4, FCz, FC2, FC4, Cz, C2, and C4) and calculated as average ERP amplitudes within latency intervals of 100 ms − 140 ms. Amplitudes of P2 were measured at the central electrodes (FC1, FCz, FC2, C1, Cz, C2, CP1, CPz, and CP2) and calculated as average ERP amplitudes within latency intervals of 200 ms − 240 ms.

#### Statistical analyses

Data analyses were performed using MATLAB R2016a. Behavioral data (intensity ratings, emotional reactions, reaction times, accuracies) and ERP data (N1 and P2 amplitudes) were analyzed using a repeated measures ANOVA of 2 “stimuli type” (painful stimuli, non-painful stimuli) × 2 “group” (High-AQ group, Low-AQ group). The within-participants’ factor was “stimuli type” (painful stimuli, non-painful stimuli), the between-participants’ factor was “group” (High-AQ group, Low-AQ group). If the interactions between the 2 factors were significant, simple effects analyses between the 2 groups were performed for each stimuli type.

## Results

### Study 1: the relationship between autistic traits and pain/non-pain sensitivity

#### Common method bias test

Since this study used a self-report form to collect data, the results may be influenced by common method bias. We used Harman’s single-factor test (54), and the results showed that the first factor under the unrotated condition explained 12.01% of the total variance, or less than 40%, suggesting there was no significant common method bias (54).

#### Descriptive and correlation analysis

**Figure 2:**
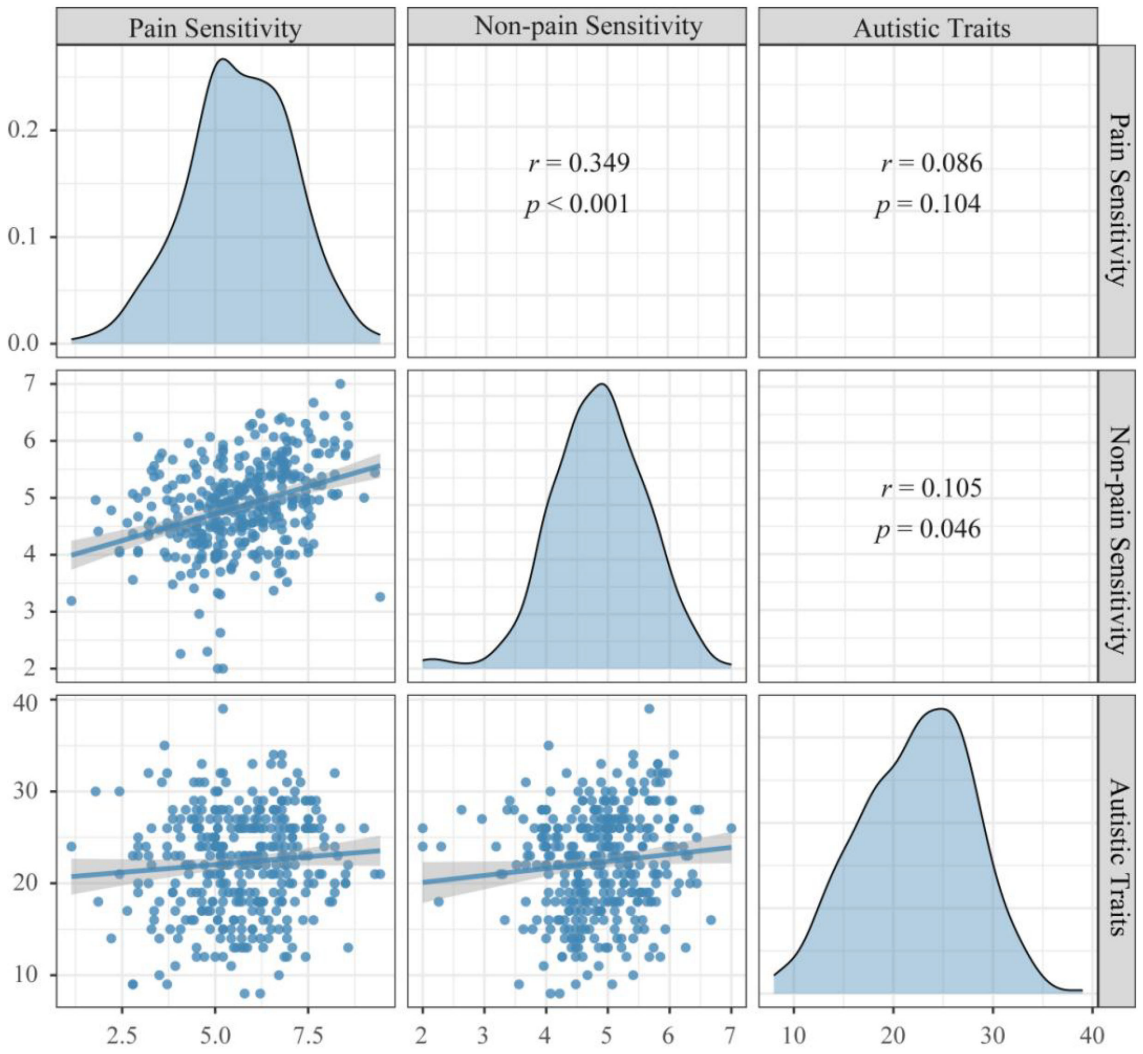
Correlation between Autistic Traits and Pain/Non-pain Sensitivity. Autistic Traits: Scores of the Autism Spectrum Quotient, Pain Sensitivity: Scores of the Pain Sensitivity Questionnaire, Non-pain Sensitivity: Scores of the Highly Sensitive Person Scale.

Results of descriptive statistics and Pearson correlation coefficients of the variables were shown in **Figure 2**. The result suggested that Autistic Traits were positively correlated with Non-pain Sensitivity (*r* = 0.105, *p* = 0.046), but not associated with Pain Sensitivity (*r* = 0.086, *p* = 0.104).

#### Differences between High-AQ and Low-AQ groups

The outcomes of the statistical analyses conducted through an independent sample *t*-test between High-AQ and Low-AQ groups are presented in **Table 2**. The High-AQ group exhibited significantly higher levels of Non-pain Sensitivity than the Low-AQ group. However, there was no significant difference in Pain Sensitivity between the High-AQ and Low-AQ groups.

**Table 2:** Group differences among variables.

	High-AQ (*n* = 35)	Low-AQ (*n* = 35)	*t*	*p*
	*M* ± *SD*	*M* ± *SD*		
Autistic Traits	30.54 ± 2.09	12.17 ± 1.85	38.88	< 0.001
Pain Sensitivity	5.88 ± 1.56	5.37 ± 1.44	1.42	0.160
Non-pain sensitivity	5.22 ± 0.66	4.77 ± 0.60	3.01	0.004

Autistic Traits: Scores of the Autism Spectrum Quotient, Pain Sensitivity: Scores of the Pain Sensitivity Questionnaire, Non-pain Sensitivity: Scores of the Highly Sensitive Person Scale.

### Study 2: cognitive-neural responses to painful and non-painful stimuli in individuals with autistic traits

#### Behavioral results

Behavioral results of reaction times, accuracies, intensity ratings, and emotional reaction ratings are summarized in **Table 3**. No significant main effect nor interaction was found for reaction times (all *ps* > 0.05).

**Table 3:** Summary of repeated-measure ANOVA results of behavioral data of Study 2.

	Intensity ratings			Emotional reactions			Reaction times			Accuracies		
	*F*	*p*	η_p_^2^	*F*	*p*	η_p_^2^	*F*	*p*	η_p_^2^	*F*	*p*	η_p_^2^
stimuli type	**537.83**	**< 0.001**	**0.89**	**63.05**	**< 0.001**	**0.50**	1.20	0.278	0.02	**24.35**	**< 0.001**	**0.28**
group	**8.37**	**0.005**	**0.12**	**4.12**	**0.047**	**0.06**	0.28	0.598	< 0.01	1.98	0.164	0.03
stimuli type × group	**10.37**	**0.002**	**0.14**	**5.35**	**0.024**	**0.08**	1.20	0.284	0.02	0.11	0.738	< 0.01

Results were obtained using a two-way mixed-design ANOVA with within-participant factors of “stimuli type” (painful stimuli, non-painful stimuli) and the between-participants factor of “group” (High-AQ, Low-AQ). Significant comparisons (p < 0.05) are indicated in boldface.

Accuracies were modulated by the main effect of “stimuli type” (*F*_(1, 64)_ = 24.35, *p* < 0.001, η_p_^2 = ^0.28), indicating that the painful stimuli (87.70% ± 1.90%) were judged less accurate than the non-painful stimuli (97.50% ± 0.90%).

Intensity ratings were modulated by the main effect of “stimuli type” (*F*_(1, 64)_ = 537.83, *p* < 0.001, η_p_^2^ = 0.89) and “group” (*F*_(1, 64)_ = 8.37, *p* = 0.005, η_p_^2^ = 0.12). The intensity ratings of painful stimuli (4.81 ± 0.14) were higher than the non-painful stimuli (1.70 ± 0.08), and the High-AQ group (3.52 ± 0.13) responded with higher intensity ratings than Low-AQ group (2.99 ± 0.13). The intensity ratings were modulated by the interaction between “stimuli type” and “group” (*F*_(1, 64)_ = 10.37, *p* = 0.002, η_p_^2^ = 0.14). Simple effect analysis showed that the High-AQ group (2.18 ± 0.12) responded with higher intensity ratings than Low-AQ group (1.22 ± 0.12; *p* < 0.001) to the non-painful stimuli. However, there was no significant difference in intensity ratings between the High-AQ group (4.86 ± 0.19) and Low-AQ group (4.76 ± 0.19; *p* = 0.714) to the painful stimuli.

Emotional reactions were modulated by the main effects of “stimuli type” (*F*_(1, 64)_ = 63.05, *p* < 0.001, η_p_^2^ = 0.50) and “group” (*F*_(1, 64)_ = 4.12, *p* = 0.047, η_p_^2^ = 0.06). Participants felt more negative to the painful stimuli (5.15 ± 0.14) than non-painful stimuli (3.79 ± 0.15), and the High-AQ group (4.71 ± 0.17) felt more negative than Low-AQ group (4.23 ± 0.17). Importantly, the emotional reactions were modulated by the interaction of “stimuli type” and “group” (*F*_(1, 64)_ = 5.35, *p* = 0.024, η_p_^2^ = 0.08). Simple effect analysis showed that the High-AQ group (4.23 ± 0.22) responded more negatively than Low-AQ group (3.36 ± 0.22; *p* = 0.006) to the non-painful stimuli. However, there was no significant difference in emotional reactions between the High-AQ group (5.19 ± 0.19) and Low-AQ group (5.11 ± 0.19; *p* = 0.756) to the painful stimuli.

#### ERP results

**Figure 3:**
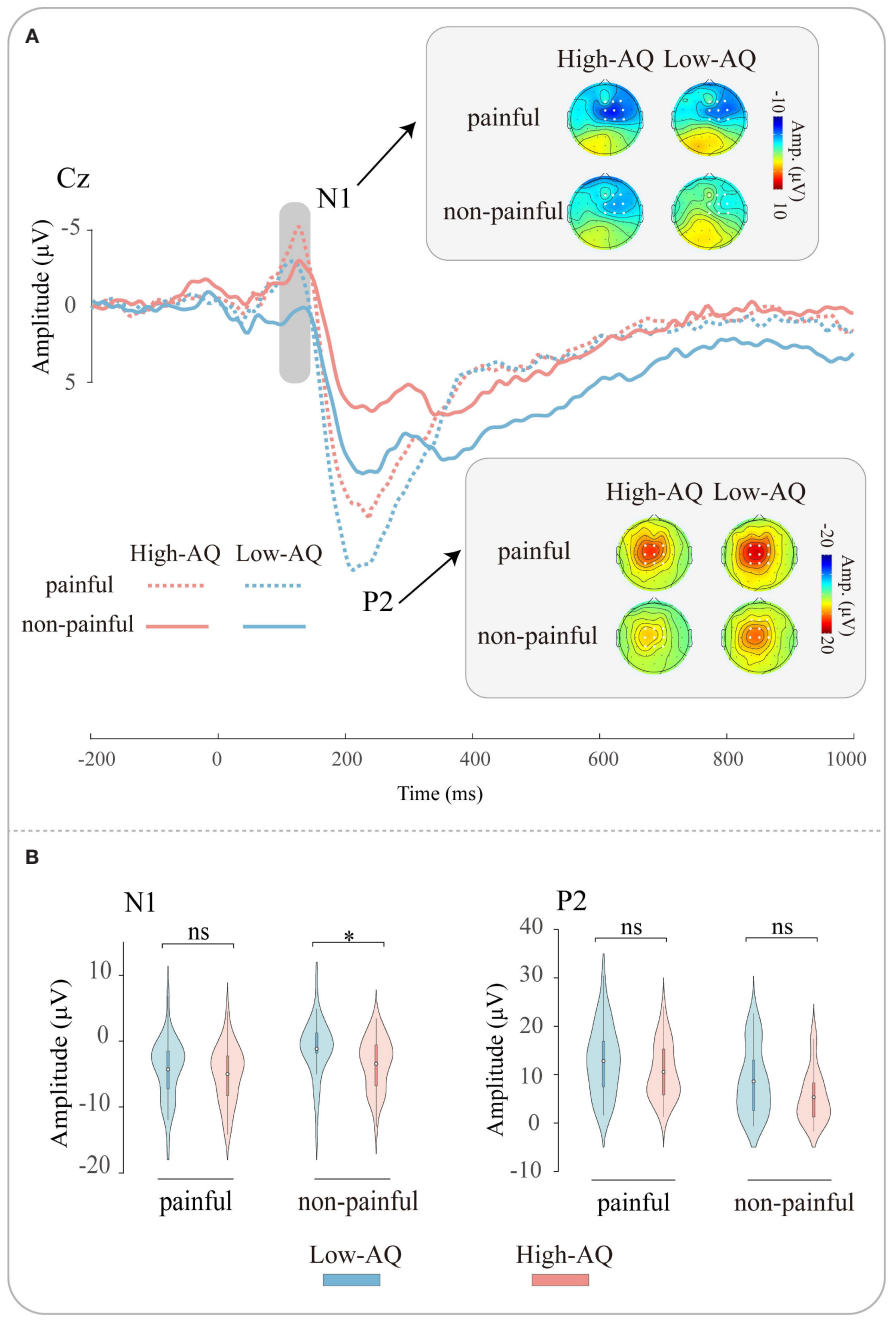
ERP waveforms and scalp topography distributions exhibited by the High-AQ (red lines) and Low-AQ (blue lines) groups in response to painful (dotted lines) and non-painful (solid lines) stimuli **(A)**. Violin plots **(B)** illustrate the interquartile ranges of the features along with the mean (white points) exhibited by the High-AQ (red) and Low-AQ (blue) groups to painful and non-painful stimuli. ns: p > 0.05, *p < 0.05.

ERP waveforms, scalp topographies, and violin plots for Study 2 are shown in **Figure 3**. Results of the statistical analyses of the ERP amplitudes are summarized in **Table 4**.

**Table 4:** Summary of statistical analyses of ERP amplitudes.

	N1			P2		
	*F*	*p*	η_p_^2^	*F*	*p*	η_p_^2^
stimuli type	**53.69**	**< 0.001**	**0.46**	**74.83**	**< 0.001**	**0.54**
group	2.15	0.148	0.03	3.57	0.063	0.05
stimuli type × group	**5.79**	**0.019**	**0.08**	0.86	0.357	0.01

Results were obtained using a two-way mixed-design ANOVA with within-participant factors of “stimuli type” (painful stimuli, non-painful stimuli) and the between-participants factor of “group” (High-AQ, Low-AQ). Significant comparisons (p < 0.05) are indicated in boldface.

N1 amplitudes were modulated by the main effect of “stimuli type” (*F*_(1, 64)_ = 53.69, *p* < 0.001, η_p_^2^ = 0.46), N1 amplitudes of painful stimuli (-4.64 μV ± 0.55 μV) were larger than non-painful stimuli (-2.29 μV ± 0.52 μV). The N1 amplitudes were modulated by the interaction of “stimuli type” and “group” (*F*_(1, 64)_ = 5.79, *p* = 0.019, η_p_^2^ = 0.08). Simple effects analysis revealed that N1 amplitudes were larger in the High-AQ group (-3.42 μV ± 0.73 μV) than in the Low-AQ group (-1.16 μV ± 0.73 μV) in response to non-painful stimuli. However, there was no difference in N1 amplitudes between High-AQ (-5.00 μV ± 0.78 μV) and Low-AQ (-4.29 μV ± 0.78 μV) groups in response to painful stimuli.

P2 amplitudes were modulated by the main effect of “stimuli type” (*F*_(1, 64)_ = 74.83, *p* < 0.001, η_p_^2^ = 0.54), painful stimuli (11.71 μV ± 0.80 μV) elicited larger P2 amplitudes than non-painful stimuli (7.00 μV ± 0.77 μV).

#### Correlation between ERP data and AQ scores

**Figure 4:**
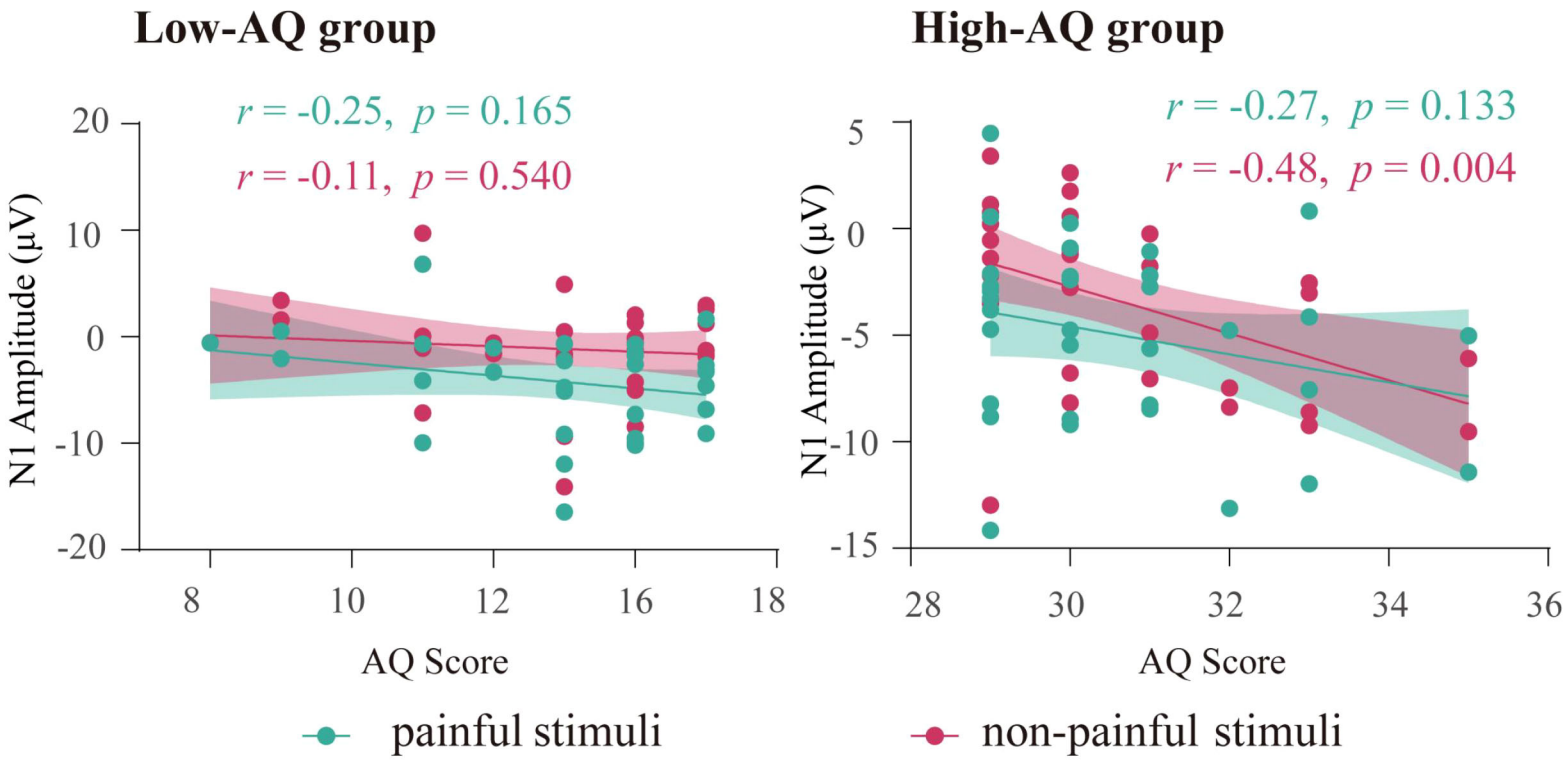
The correlation between N1 amplitudes and AQ scores. Each dot represents the value of a single participant from High-AQ (right panel) and Low-AQ (left panel) groups to painful (green) and non-painful (red) stimuli. The thick green line (painful stimuli) and red line (non-painful stimuli) indicate the best linear fit, while the shaded areas represent the 95% confidence interval.

Results showed that for High-AQ group, there was a significantly negative correlation between the AQ scores and the N1 amplitudes to non-painful stimuli. However, no significant correlation was found for Low-AQ group. No other significant correlation was found (all *ps* > 0.05). The correlation results are displayed in **Figure 4**.

## Discussion

This research explored the painful and non-painful sensation and their cognitive-neurological mechanisms of individuals with autistic traits through questionnaires (Study 1) and experimental (Study 2) methodologies. The results from Study 1 indicated that Autistic Traits were positively correlated with Non-pain Sensitivity, but not associated with Pain Sensitivity. The results from Study 2 indicated that the High-AQ group showed higher intensity ratings, more negative emotional reactions, and larger N1 amplitudes than the Low-AQ group in response to the non-painful stimuli, but no difference was found between High-AQ and Low-AQ groups in response to painful stimuli. These findings suggest that individuals with autistic traits may experience enhanced non-painful sensation but intact painful sensation.

In line with previous studies (7, 10–13, 25, 55), the AQ questionnaire was used to select participants in the present study, participants with high scores on the AQ questionnaire (High-AQ group) were considered as individuals with autistic traits. Consistent with previous studies (9, 12, 25, 56, 57), the present study also found the High-AQ group responded with higher intensity ratings and more negative emotional reactions than the Low-AQ group to the stimuli. These findings indicated that individuals with autistic traits not only perceived sensory stimuli as more intense but also exhibited more negative emotional responses, potentially reflecting their heightened sensory sensitivity and emotional reactivity associated with ASD.

In line with previous studies (41, 47, 58–61), Study 2 documented the enlarged N1 and P2 amplitudes for painful stimuli compared to non-painful stimuli. Both ERP components have been identified as independent markers of neural reactions to the electrical stimuli (6, 44, 62–66). The N1 component is commonly associated with initial sensory processing and the P2 component pertains to the cognitive evaluation of sensory information of the electrical stimuli (12, 21, 50, 67, 68). The ERP results in this study suggested that painful stimuli capture more mental resources of sensory processing and cognitive evaluation than non-painful stimuli. These ERP results were also consistent with the behavioral results, which showed that participants responded to painful stimuli with less accurate judgment, higher intensity ratings, and more negative emotional reactions than non-painful stimuli.

Interestingly, both behavioral and ERP responses in Study 2 were modulated by the interaction between “stimuli type” and “group”. For the non-painful stimuli, the High-AQ group responded with higher intensity ratings, more negative emotional reactions, and larger N1 amplitudes than the Low-AQ group. However, there was no difference in response to painful stimuli between the High-AQ and Low-AQ groups. In addition, N1 amplitudes of non-painful stimuli were negatively correlated with the High-AQ group’s AQ scores, that is, participants in the High-AQ group with higher AQ scores tended to exhibit larger N1 amplitudes in response to non-painful stimuli. As the N1 component is commonly associated with initial sensory processing (12, 50, 66, 69–71), these results suggest that the High-AQ group may have intact processing to painful stimuli but enhanced sensitivity to non-painful stimuli.

Further supporting these findings, our questionnaire survey in Study 1 indicated a positive correlation between Autistic Traits and Non-pain Sensitivity, as well as higher levels of Non-pain Sensitivity for High-AQ group than the Low-AQ group. This aligns with the notion that individuals with autistic traits perceive and process sensory information from their environment more intensively, leading to a greater complexity in sensory processing (56, 72). However, there was no correlation between Autistic Traits and Pain Sensitivity, nor difference in Pain Sensitivity between the High-AQ and Low-AQ groups, echoing the ERP findings in Study 2 and aligning with previous research (17, 25, 73, 74). This suggests a distinct sensory profile associated with ASD, where increased sensory sensitivity does not extend uniformly to all sensory modalities, particularly in the context of painful sensation.

This observation can be particularly be explained by some theories. Based on the Enhanced Perceptual Functioning model (26), individuals with ASD may excel in processing fine-grained details and patterns in their environment, thereby exhibiting superior performance in certain sensory and perceptual tasks, and have heightened perceptual sensitivity compared to neurotypical individuals. This theory suggests that individuals with autistic traits may also experience enhanced non-painful sensitivity because of their superior performance in non-painful sensory. In addition, according to the Bayesian Model’s Prior Hypothesis (75), individuals with ASD may possess less established or weaker prior expectations about sensory events. This would mean that sensory processing of individuals with autistic traits may rely more heavily on incoming sensory information rather than on preconceived notions or predictions, leading to an amplified response to non-painful stimuli. On the other hand, the intact painful sensation of High-AQ group may be explained by the fact that painful stimuli were highly salient and potentially evolutionary significant, both the High-AQ and Low-AQ groups might share similar strong priors regarding pain due to its critical nature for survival, leading to a uniform sensation and neural response across individuals, regardless of their autistic traits.

Despite the methodological rigor with which these studies were conducted, several limitations should be acknowledged. Firstly, the Highly Sensitive Person Scale and the Pain Sensitivity Questionnaire used in Study 1 had a few overlaps in content. The Highly Sensitive Person Scale encompasses a broader range of sensitivity experiences, including sensitivity to emotions, environments, and physical stimuli, whereas the Pain Sensitivity Questionnaire focuses solely on sensitivity to physical pain. Notably, the Highly Sensitive Person Scale also includes one question regarding pain sensitivity, which may limit our understanding of the relationship between autistic traits and these sensitivity experiences when comparing results directly from the Highly Sensitive Person Scale and Pain Sensitivity Questionnaire. This limitation suggests that future research should consider employing more specialized and nuanced measures to assess different types of sensitivity experiences. Secondly, while individuals with autistic traits and individuals with ASD may exhibit similar behaviors in certain domains, a high score on the AQ does not necessarily equate to a clinical diagnosis of ASD (76–78). This distinction creates a significant gap between the manifestation of autistic traits in the general population and in individuals with ASD. Therefore, our findings based on AQ scores may not fully generalize to the broader ASD population, thus limiting the applicability of our results to clinical contexts. Finally, the design of this study is cross-sectional, capturing only a single point in time. Autistic traits and sensory sensitivities may evolve over time or in different contexts. Longitudinal research is needed to understand the developmental trajectory of these characteristics and their impact on individuals’ lives.

In summary, this study explored the painful and non-painful sensation in individuals with autistic traits, founding an enhanced sensation to non-painful stimuli but not to painful stimuli. These findings enrich our understanding of the sensory experiences in autism spectrum characteristics, suggesting a differential cognitive-neural processing mechanism that might exist between painful and non-painful sensation in individuals with autistic traits. These insights not only contribute to our theoretical knowledge but may also inform practical approaches to support individuals with ASD in managing sensory experiences in their daily lives.

## Data availability statement

The original contributions presented in the study are included in the article/supplementary material. Further inquiries can be directed to the corresponding author.

## Ethics statement

The studies involving humans were approved by Chongqing Normal University research ethics committee. The studies were conducted in accordance with the local legislation and institutional requirements. The participants provided their written informed consent to participate in this study.

## Author contributions

HQ: Conceptualization, Data curation, Formal analysis, Investigation, Methodology, Software, Visualization, Writing – original draft, Writing – review & editing. MS: Conceptualization, Data curation, Formal analysis, Methodology, Software, Writing – review & editing. ZW: Conceptualization, Data curation, Methodology, Software, Writing – review & editing. YZ: Conceptualization, Methodology, Writing – review & editing. SL: Conceptualization, Methodology, Writing – review & editing. LC: Investigation, Methodology, Writing – review & editing. JM: Conceptualization, Funding acquisition, Methodology, Project administration, Resources, Supervision, Visualization, Writing – review & editing.
